# Patient acceptance and implementation of automated breast ultrasound in intensified breast cancer surveillance

**DOI:** 10.1007/s00404-026-08603-z

**Published:** 2026-11-13

**Authors:** Maria Eleni Hatzipanagiotou, Kathrin Hartwig, Miriam Fernandez-Pacheco, Sara Plavic-Radeka, Jonas Roth, Ivana Holzhauser, Simone Hammer, Olaf Ortmann, Stephan Seitz

**Affiliations:** 1 Department of Gynecology and Obstetrics, University Medical Center Regensburg, Comprehensive Cancer Center (CCC) Würzburg, Erlangen, Regensburg, Augsburg (WERA), National Center for Tumor Diseases (NCT), Regensburg, Germany; 2 Institute of Clinical Human Genetics, University Hospital Regensburg, Regensburg, Germany; 3 Department of Radiology, University Hospital Regensburg, Regensburg, Germany

**Keywords:** Automated breast ultrasound, Intensified breast cancer surveillance, Surveillance program, ABUS, German Consortium for Hereditary Breast and Ovarian Cancer

## Abstract

**Purpose:**

Automated breast ultrasound (ABUS) is a standardized ultrasound technique that may reduce operator dependency compared with hand-held ultrasound. Evidence on patient acceptance and implementation in intensified breast cancer surveillance remains limited. This study evaluated patient acceptance and clinical implementation of ABUS within a certified center of the German Consortium for Hereditary Breast and Ovarian Cancer.

**Methods:**

In this prospective single-center observational study, women enrolled in an intensified breast cancer surveillance program underwent ABUS examinations and completed standardized questionnaires. Patient-reported outcomes included examination-related discomfort, comparison with mammography, willingness to repeat the examination, and willingness to recommend ABUS. Workflow parameters and changes in acquisition time over repeated examinations were assessed prospectively.

**Results:**

73 women contributed 162 ABUS examinations and corresponding questionnaires. Of these, 46/73 (63.0%) carried pathogenic variants in BRCA1, BRCA2, PALB2, or TP53, and 27/73 (37.0%) had a previous breast cancer diagnosis. Median pain score was 0 (IQR 0–1) for both breasts. Among women with previous mammography and an available comparative rating, 56/57 (98.2%) rated ABUS as more comfortable. All 73 participants (100%) were willing to undergo future ABUS examinations and recommend the procedure. Mean acquisition and interpretation times were 31.9 ± 6.2 and 9.95 ± 4.0 min, respectively. Acquisition time decreased significantly with increasing examination number (*β* = − 1.72 min/examination; *p* < 0.001).

**Conclusions:**

ABUS was associated with high reported patient acceptance and was feasible to integrate into intensified breast cancer surveillance, with improving acquisition efficiency over time.

## What does this study adds to the clinical work

**Table:** 

Automated breast ultrasound (ABUS) is a well-accepted and feasible imaging modality for intensified hereditary breast cancer surveillance, with minimal patient discomfort and high willingness to undergo repeat examinations. Its integration into routine clinical workflows is supported by increasing efficiency with repeated use, although its diagnostic value within multimodal surveillance strategies requires further evaluation.	

## Introduction

Women carrying pathogenic variants in breast cancer susceptibility genes, such as *BRCA1, BRCA2, PALB2, CHEK2, ATM*, or *TP53*, are at substantially increased lifetime risk of developing breast cancer and therefore require tailored surveillance strategies aimed at early detection [1, 2]. Recent large-scale genetic studies have further refined risk estimates for hereditary breast cancer susceptibility genes and reinforced the need for individualized, risk-adapted surveillance strategies [1–3]. In Germany, women at increased breast cancer risk are enrolled in structured surveillance programs coordinated by the German Consortium for Hereditary Breast and Ovarian Cancer (GC-HBOC). These programs combine different imaging modalities according to age, genetic background, and individual risk profile. Current consensus recommendations include annual magnetic resonance imaging (MRI) as the central imaging modality, complemented by mammography and ultrasound depending on patient age and clinical circumstances [3, 4]. Long-term data from the GC-HBOC surveillance program have demonstrated the effectiveness and clinical feasibility of intensified surveillance strategies in this high-risk population [5]. Despite continuous improvements in breast imaging, early detection remains challenging in women with dense breast tissue. Increased mammographic density is associated with both an elevated breast cancer risk and reduced mammographic sensitivity due to the masking effect of fibroglandular tissue [6, 7]. Consequently, considerable efforts have been directed toward the evaluation of supplemental imaging techniques capable of improving lesion detection in women with dense breasts and in high-risk populations [4, 6]. Breast ultrasound represents an important adjunct to mammography because it is widely available, free of ionizing radiation, and capable of detecting lesions that may not be visible on mammography. However, hand-held ultrasound (HHUS) is highly operator-dependent and subject to substantial variability in image acquisition, documentation, and interpretation. These limitations may affect reproducibility and complicate quality assurance, particularly in longitudinal surveillance programs requiring repeated examinations over many years [8, 9].

Automated breast ultrasound (ABUS) was developed to address some of the limitations of HHUS by separating image acquisition from image interpretation and by providing standardized three-dimensional image datasets of the entire breast [10, 11]. Standardized acquisition protocols allow image acquisition to be performed by specially trained medical assistants, while image interpretation can subsequently be carried out by physicians. In addition, a retrospective review of complete image volumes may improve reproducibility and facilitate quality control [12]. Previous studies have shown that supplemental ABUS can increase cancer detection in women with dense breasts although this benefit may be accompanied by increased recalls and false-positive findings [10, 13–15]. Evidence in women undergoing intensified high-risk surveillance is more limited; notably, Van Zelst et al. found no additional cancer detection when ABUS was added to MRI and mammography in BRCA1/2 mutation carriers [16].

While diagnostic performance has been extensively investigated, considerably less attention has been paid to patient-centered outcomes and practical implementation. Successful surveillance programs depend not only on diagnostic accuracy but also on patient acceptance, workflow integration, resource utilization, and long-term adherence. This aspect may be particularly relevant in intensified breast cancer surveillance, where women undergo repeated imaging examinations over many years. Previous studies have reported favorable patient experiences with ABUS and suggested high acceptance among screened women [17]. However, data specifically addressing patient acceptance and implementation within intensified breast cancer surveillance programs remain limited. Therefore, the aim of the present study was to evaluate patient acceptance and clinical implementation of ABUS within a certified intensified breast cancer surveillance program. In addition to patient-reported outcomes, organizational aspects, workflow characteristics, and implementation-related parameters were assessed to provide real-world evidence regarding the feasibility of integrating ABUS into routine intensified breast cancer surveillance.

## Methods

### Study design and setting

This prospective observational study was conducted at the Center for Familial Breast and Ovarian Cancer Regensburg, a certified center of the GC-HBOC. The study was designed to evaluate patient acceptance and clinical implementation of ABUS within the established intensified breast cancer surveillance program. ABUS examinations were integrated into routine clinical surveillance procedures, and patient-reported outcomes as well as workflow-related parameters were prospectively collected throughout the study period. This study was approved by the Ethics Committee of the University of Regensburg (reference number 23–3282-101). All participants provided written informed consent prior to study inclusion.

### Study population

Women participating in the intensified breast cancer surveillance program at the study center were eligible for inclusion. Eligibility followed the risk-adapted surveillance criteria of the GC-HBOC and comprised three groups: (1) carriers of pathogenic variants in high-risk breast cancer susceptibility genes (BRCA1, BRCA2, PALB2, or TP53); (2) carriers of pathogenic variants in other breast cancer susceptibility genes included in the intensified surveillance program (ATM, BARD1, BRIP1, CDH1, CHEK2, RAD51C, RAD51D, PTEN, or STK11); and (3) women without an identified pathogenic variant in these genes who had a sufficiently elevated familial breast cancer risk, as determined using a computer-based risk assessment model, to qualify for intensified surveillance. Women were eligible irrespective of whether they were unaffected or had a previous diagnosis of breast cancer; a personal history of breast cancer could occur within any of the eligibility groups and was therefore recorded separately. Women presenting for their scheduled breast ultrasound examination within the intensified breast cancer surveillance program were invited to participate in the study. Participation consisted of undergoing an additional ABUS examination and completing the study-specific questionnaires. Participation was voluntary and had no influence on routine clinical management or surveillance recommendations. The additional ABUS examination was performed during the same surveillance visit as the scheduled routine HHUS examination. In women carrying pathogenic variants in BRCA1, BRCA2, PALB2, or TP53, routine breast ultrasound was scheduled every six months. Women carrying pathogenic variants in ATM, BARD1, BRIP1, CDH1, CHEK2, RAD51C, RAD51D, PTEN, or STK11 underwent annual routine breast ultrasound. Women without an identified pathogenic variant but meeting high-risk criteria based on individualized risk assessment likewise underwent annual routine breast ultrasound. MRI and mammography continued according to the established risk-adapted GC-HBOC surveillance protocol and were not altered by study participation.

### ABUS acquisition and image interpretation

**Fig. 1:**
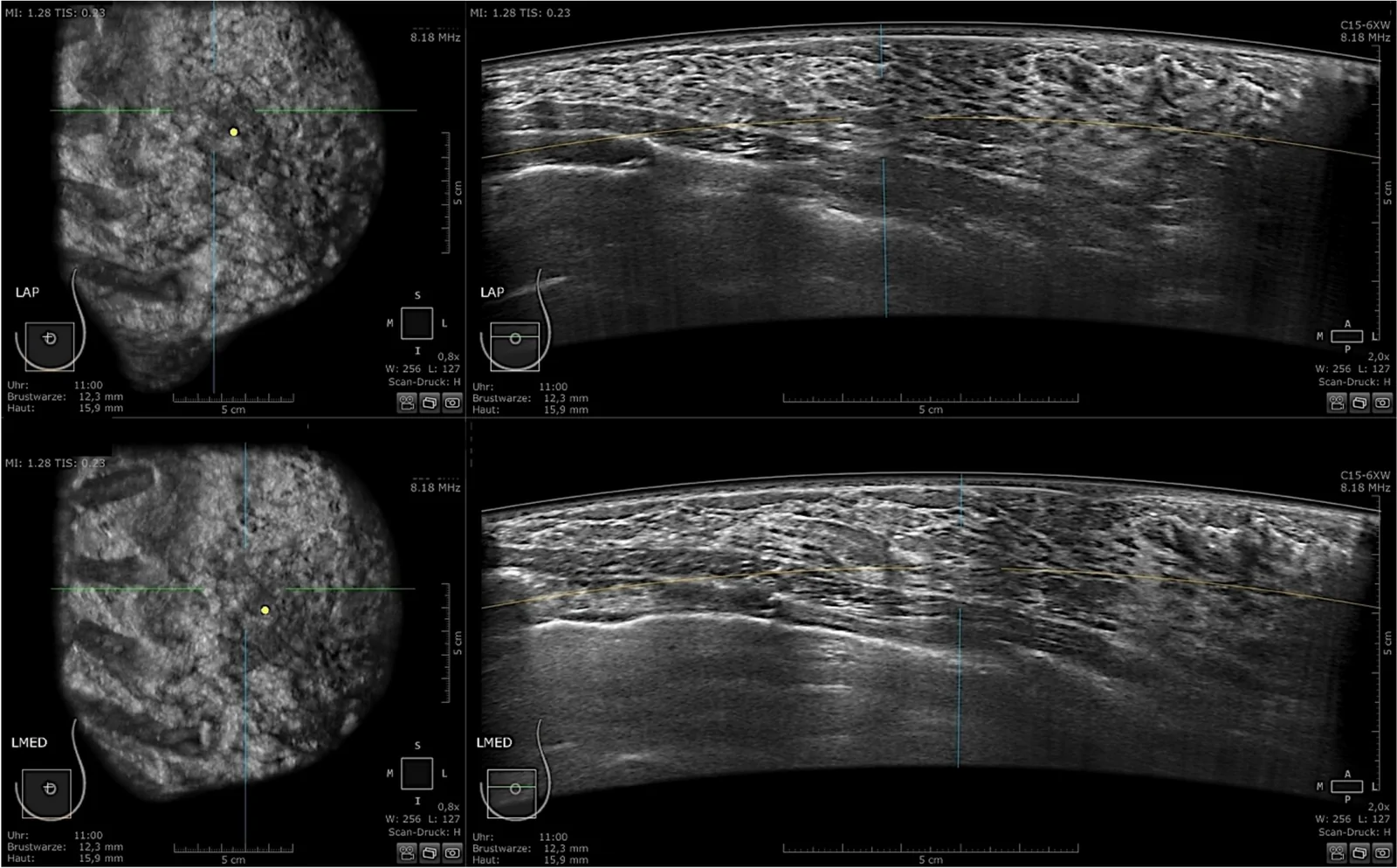
Representative ABUS images of the left breast obtained in the central (upper row) and medial (lower row) scanning positions. Coronal reconstructions are shown on the left and corresponding transverse images on the right

ABUS examinations were performed using the Invenia™ Automated Breast Ultrasound System (GE HealthCare, Chicago, IL, USA). Image acquisition was conducted by specially trained medical assistants according to a standardized institutional protocol. Patients were examined in the supine position, and at least three acquisitions were obtained per breast depending on breast size and anatomy to ensure complete coverage of the breast tissue. Acquired image volumes were transferred to dedicated workstations for subsequent interpretation. Image acquisition and image interpretation were performed independently. Each ABUS examination was interpreted by one of four physicians specifically trained in ABUS and experienced in breast imaging. No artificial intelligence (AI), computer-aided detection (CAD), or other automated image-analysis software was used for image interpretation. All examinations were reviewed using dedicated ABUS workstations allowing multiplanar reconstruction and evaluation of the complete three-dimensional image dataset. Interpretation was performed as part of routine clinical surveillance. The present study was not designed to evaluate diagnostic performance, and therefore no analyses of sensitivity, specificity, or cancer detection rates were performed. Figure 1 shows representative ABUS images of the left breast acquired in the central and medial scanning positions.

### Patient questionnaire

Immediately after completion of the examination, participants were asked to complete a standardized questionnaire developed specifically for this implementation study. The questionnaire assessed patient acceptance, examination-related discomfort, overall satisfaction with the examination, comparison of ABUS with mammography, willingness to undergo future ABUS examinations, and willingness to recommend the procedure to other women. Additional questions addressed waiting times, communication preferences regarding examination results, and satisfaction with information provided before the examination. Pain perception was assessed using a visual analog scale (VAS) ranging from 0 to 10, with higher scores indicating greater examination-related discomfort.

### Workflow assessment

Implementation and workflow characteristics were prospectively documented throughout the study period. Emphasis was placed on the practical integration of ABUS into the existing intensified breast cancer surveillance program. Examination duration, interpretation time, waiting time for appointments, technical recall examinations, and organizational aspects of care were systematically recorded. In addition, patient evaluations of the examination process and the performance of the medical assistants conducting the examinations were assessed. To investigate implementation effects over time, repeated examinations were analyzed longitudinally.

### Statistical analysis

Statistical analyses were performed using IBM SPSS Statistics version 31.0 (IBM Corp., Armonk, NY, USA). Continuous variables are presented as mean ± standard deviation (SD) or median and interquartile range (IQR), as appropriate, whereas categorical variables are reported as absolute frequencies and percentages. Distribution of continuous variables was assessed descriptively before summary statistics were selected. Baseline and patient-reported outcomes were analyzed at the participant level (*n* = 73). For descriptive patient-reported outcomes, only the first questionnaire completed by each participant was included. Longitudinal changes in acquisition time were analyzed using a linear mixed-effects model with a patient-specific random intercept. Interpretation times across repeated examinations were analyzed descriptively. Item-specific denominators were reported where applicable. Results are reported as regression coefficients (β) with corresponding 95% confidence intervals (CI). All analyses were exploratory in nature. No formal a priori sample-size calculation was performed because the study was designed as an exploratory implementation study. A two-sided p-value of less than 0.05 was considered statistically significant.

## Results

### Study population

A total of 73 unique women participated in the study and contributed 162 ABUS examinations with corresponding questionnaires. 27 women contributed one examination, 23 contributed two, 12 contributed three, four contributed four, five contributed five, and two contributed six examinations. Patient-reported outcomes were analyzed at the participant level (*n* = 73), whereas repeated observations were used for longitudinal analyses. Of the 73 women 46/73 (63.0%) carried a pathogenic variant in BRCA1, BRCA2, PALB2, or TP53, 14/73 (19.2%) carried a pathogenic variant in another breast cancer susceptibility gene included in the intensified surveillance program, and 13/73 (17.8%) had no identified pathogenic variant but qualified for intensified surveillance based on elevated familial breast cancer risk. Overall, 27/73 women (37.0%) had a personal history of breast cancer. Among the 27 women with a personal history of breast cancer, 15/27 (55.6%) carried a pathogenic variant in BRCA1, BRCA2, PALB2, or TP53, 6/27 (22.2%) carried a pathogenic variant in another breast cancer susceptibility gene included in the intensified surveillance program, and 6/27 (22.2%) fulfilled familial high-risk criteria without an identified pathogenic variant. The median age was 46 years (range 25–71 years). Approximately one-third of participants had previously undergone an ABUS examination. Detailed baseline characteristics are summarized in Table 1.

**Table 1:** Patient characteristics

Characteristic	Women (*n* = 73)
Number of ABUS examinations/questionnaires	162
Age, years, median (range)	46 (25–71)
Eligibility for intensified breast cancer surveillance	
Pathogenic variant in BRCA1, BRCA2, PALB2, or TP53	46/73 (63.0%)
BRCA1	20/73 (27.4%)
BRCA2	24/73 (32.9%)
PALB2	2/73 (2.7%)
TP53	0/73 (0%)
Other pathogenic variants included in the surveillance program	14/73 (19.2%)
ATM	0/73 (0%)
BARD1	2/73 (2.7%)
BRIP1	0/73 (0%)
CDH1	1/73 (1.4%)
CHEK2	5/73 (6.8%)
RAD51C	3/73 (4.1%)
RAD51D	2/73 (2.7%)
PTEN	1/73 (1.4%)
STK11	0/73 (0%)
Familial high-risk criteria without an identified pathogenic variant	13/73 (17.8%)
Personal history of breast cancer	
Previous breast cancer	27/73 (37.0%)
BRCA1, BRCA2, PALB2, or TP53 group	15/27 (55.6%)
Other pathogenic variant group	6/27 (22.2%)
Familial high-risk group without identified pathogenic variant	6/27 (22.2%)
No previous breast cancer	46/73 (63.0%)
Previous experience with ABUS	
Previous ABUS examination	26/73 (35.6%)
First ABUS examination	46/73 (63.0%)
Missing information	1/73 (1.4%)

Baseline characteristics of the study population participating in the intensified breast cancer surveillance program. Data are presented as n/N (%) unless otherwise specified

### Patient acceptance

Overall patient acceptance of ABUS was high. Examination-related discomfort was minimal, with median pain scores of 0 (IQR 0–1) for both breasts. No pain was reported by 50/71 women (70.4%) for the left breast and 51/69 (73.9%) for the right breast. Among 57 women who had previously undergone mammography and provided a comparative rating, 56/57 (98.2%) rated ABUS as more comfortable, 1/57 (1.8%) rated both examinations as comparable, and none rated ABUS as less comfortable. This comparison reflected participant-reported preference and was not based on a standardized comparative discomfort assessment. Fourteen women had never undergone mammography and two did not provide a response. Importantly, all participants stated that they would undergo the examination again if recommended and would recommend the procedure to other women. Furthermore, 70/72 participants (97.2%) considered ABUS to be an important component of their surveillance program. Patient-reported outcomes are summarized in Table 2.

**Table 2:** Patient-reported acceptance of automated breast ultrasound (ABUS)

Parameter	Result
Examination-related discomfort Pain score, left breast, median (IQR)	0 (0–1), *n* = 71
Pain score, right breast, median (IQR)	0 (0–1), *n* = 69
No pain (VAS 0), left breast	50/71 (70.4%)
No pain (VAS 0), right breast	51/69 (73.9%)
Comparison with mammography	
ABUS rated more comfortable*	56/57 (98.2%)
ABUS and mammography rated comparable*	1/57 (1.8%)
ABUS rated less comfortable*	0/57 (0%)
No previous mammography	14/73 (19.2%)
Missing response	2/73 (2.7%)
Overall acceptance and perceived importance	
Willingness to undergo ABUS again	73/73 (100%)
Willingness to recommend ABUS	73/73 (100%)
ABUS considered very important	70/72 (97.2%)
ABUS considered less important	2/72 (2.8%)

Data are presented as n/N (%) unless otherwise specified. Item-specific denominators reflect available responses. *The comparison with mammography includes only women with previous mammography who provided a comparative rating (*n* = 57). VAS, visual analog scale; IQR, interquartile range

### Workflow and implementation

ABUS was successfully integrated into the existing intensified breast cancer surveillance program. The median waiting time for an appointment was 0 weeks (IQR 0–1), with 60/73 participants (82.2%) reporting no waiting time. Information provided before the examination was rated positively by 72/73 participants (98.6%), and the examination performed by trained medical assistants was rated positively by 73/73 (100%). Among 71 participants with available responses, 17/71 (23.9%) preferred direct personal communication of examination results, 24/71 (33.8%) preferred written communication, and 30/71 (42.3%) considered the absence of subsequent contact to indicate a normal examination result. The mean ABUS acquisition time was 31.9 ± 6.2 min, while mean image interpretation time was 9.95 ± 4.0 min. No technical recall examinations due to inadequate image quality were required during the study period. Workflow and implementation outcomes are summarized in Table 3.

**Table 3:** Workflow and implementation outcomes

Parameter	Result
Patient-reported organizational outcomes	
Waiting time, mean ± SD, weeks	0.84 ± 2.39
Waiting time, median (IQR), weeks	0 (0–1)
No waiting time	60/73 (82.2%)
Information provided before examination rated good	72/73 (98.6%)
Examination setting rated good	68/73 (93.2%)
Examination performed by medical assistant rated good	73/73 (100%)
Preferred communication of examination results	
Personal discussion preferred	17/71 (23.9%)
Written communication preferred	24/71 (33.8%)
No contact interpreted as a normal result	30/71 (42.3%)
Missing response	2/73 (2.7%)
Workflow parameters	
ABUS acquisition time, mean ± SD, min	31.9 ± 6.2
Image interpretation time, mean ± SD, min	9.95 ± 4.0
Recall examinations	0/73 (0%)

Data refer to the initial documented examination of the 73 study participants. Categorical data are presented as n/N (%), with item-specific denominators where responses were missing. IQR, interquartile range; SD, standard deviation

### Longitudinal analysis

Among the 73 participants, 46 women underwent at least one additional ABUS examination. The linear mixed-effects analysis of acquisition time included 73 women contributing 161 valid acquisition-time measurements across up to six examination time points. For descriptive presentation, results are shown for examinations 1–5; two sixth examinations were documented in the overall cohort but were not included in the descriptive time-point summaries below.

#### Acquisition time

For the descriptive time-point analysis, 73, 46, 23, 11, and 7 acquisition-time observations were available at examinations 1–5, respectively. Mean acquisition time was 31.9 ± 6.2 min at the initial examination and decreased to 25.0 ± 6.1 min at the second examination. Mean acquisition times at examinations 3, 4, and 5 were 27.2 ± 5.4, 30.0 ± 6.3, and 23.6 ± 2.4 min, respectively. Linear mixed-effects analysis showed a significant decrease in acquisition time with increasing examination number (*β* = − 1.72 min per examination; 95% CI − 2.64 to − 0.81; *p* < 0.001).

#### Interpretation time

For interpretation time, 73 women contributed 159 observations across examinations 1–5 (73, 45, 23, 11, and 7, respectively). Mean interpretation time was 9.95 ± 4.01 min at the initial examination and decreased to 7.42 ± 2.83 min at the second examination. Mean interpretation times at examinations 3, 4, and 5 were 6.65 ± 2.42, 7.09 ± 2.26, and 8.14 ± 3.08 min, respectively.

## Discussion

In this prospective single-center study, we evaluated the clinical implementation and patient acceptance of ABUS within a certified center of the GC-HBOC providing intensified breast cancer surveillance. Patient-reported outcomes also included comparison with previous mammography experience. Overall, ABUS was associated with high reported patient acceptance, favorable organizational characteristics and increasing workflow efficiency over time. Examination-related discomfort was minimal, with median pain scores of 0 (IQR 0–1) for both breasts. All participants indicated that they would undergo the examination again and would recommend it to other women. Because participation was voluntary, the high levels of reported acceptance and willingness to undergo repeat ABUS examinations may partly reflect selection bias, as women with a more favorable attitude toward additional breast imaging may have been more likely to participate. Therefore, these findings should not be interpreted as representative of all women undergoing intensified breast cancer surveillance. Patient acceptance represents an important but often underappreciated component of surveillance programs. Women at increased risk undergo repeated imaging examinations over many years, making long-term adherence essential for surveillance effectiveness. Although long-term adherence was not assessed in the present study, high patient satisfaction may contribute to sustained participation in surveillance programs. Similar observations were reported by Mussetto et al., who found that both ABUS and HHUS were generally well accepted, although HHUS was perceived as less painful than ABUS [17]. The comparison with mammography in the present study should be interpreted as a participant-reported preference rather than as a standardized head-to-head assessment of examination-related discomfort. While previous studies have primarily focused on a screening general population undergoing supplemental breast imaging [10, 15, 18], our data extend these observations to a population undergoing intensified breast cancer surveillance.

Beyond patient acceptance, the present study also demonstrated favorable organizational characteristics of ABUS implementation. Patient satisfaction regarding organizational aspects of care was high, and no technical recall examinations occurred during the study period. Waiting times were short, and nearly all participants rated the information provided before the examination positively. Furthermore, all participants evaluated the examination performed by trained medical assistants favorably. One of the principal conceptual advantages of ABUS is the separation of image acquisition and image interpretation. Unlike HHUS, which requires direct physician involvement during image acquisition, ABUS allows image acquisition to be delegated to specially trained personnel. This workflow may improve resource allocation and increase standardization of image acquisition. Boca Bene et al. emphasized workflow standardization and reproducibility as major advantages of ABUS and discussed its potential to optimize clinical workflows through the separation of image acquisition and interpretation [9]. In our cohort, this workflow was feasible and well accepted. The observed mean acquisition time was approximately 32 min and the mean interpretation time approximately 10 min. HHUS examination and interpretation times were not prospectively recorded in the present study, precluding a direct within-cohort comparison. In published comparative studies, ABUS generally required a longer total examination time than HHUS but reduced direct physician involvement because image acquisition could be delegated to trained personnel. Brunetti et al. [19] reported a mean total examination and reading time of approximately 17 min for ABUS compared with 5 min for HHUS, whereas Güldoğan et al. [20] found that ABUS required approximately 11 min in total, including about 7 min for image acquisition and 4 min of physician interpretation. These findings suggest that the potential workflow advantage of ABUS lies less in reducing total examination time than in shifting image acquisition away from physician time. The availability of complete three-dimensional ABUS datasets may also facilitate future AI-assisted interpretation, potentially further reducing physician reading time; however, AI-assisted interpretation was not evaluated in the present study and remains outside its scope. Although Huppe et al. reported substantially shorter interpretation times of less than 3 min per examination [12], direct comparisons are difficult because of differences in study design, reader experience and clinical workflow, including the implementation of ABUS within a specialized intensified breast cancer surveillance program. Furthermore, a relevant proportion of our cohort had a personal history of breast cancer, which may have increased interpretation complexity and contributed to longer reading times. The present study demonstrated a significant reduction in examination duration over time. Examination time decreased by 1.72 min per examination (95% CI –2.64 to –0.81; *p* < 0.001), indicating a measurable learning effect. This observation is particularly relevant because implementation studies frequently focus on static workflow measurements while neglecting changes occurring during the adoption process. Our findings suggest that initial workflow assessments may overestimate long-term resource requirements. Increasing familiarity with patient positioning, acquisition protocols, and workflow organization likely contributed to the observed efficiency gains. Interpretation times were also descriptively lower at repeated examinations than at the initial examination; however, no longitudinal mixed-effects model of absolute interpretation time was performed, and these findings should therefore be interpreted descriptively. Previous reviews have suggested that ABUS may facilitate standardized workflows, but prospective data documenting implementation-related learning effects remain limited [8, 9, 18].

The present study was designed to assess patient acceptance and workflow implementation and provides no evidence regarding the diagnostic performance of ABUS or its incremental diagnostic value over MRI or HHUS. Accordingly, the favorable acceptance and workflow findings should not be interpreted as evidence supporting diagnostic superiority or replacement of established imaging modalities. Nevertheless, the present findings should be considered alongside previous studies investigating the diagnostic performance and clinical applications of ABUS in breast cancer screening and surveillance. Several studies have demonstrated that supplemental ABUS can improve breast cancer detection in women with dense breast tissue. In the large prospective SomoInsight study, Brem et al. reported an additional cancer detection yield of 1.9 cancers per 1,000 women screened when ABUS was added to mammography. This gain in cancer detection was, however, accompanied by a substantial increase in recalls: the recall rate increased from 150.2 per 1,000 women (15.0%) with mammography alone to 284.9 per 1,000 women (28.5%) with the combined imaging approach. In addition, the biopsy rate increased by 36.0 per 1,000 women screened [13]. Thus, the additional diagnostic yield of ABUS in this population was accompanied by a relevant increase in additional diagnostic work-up. Comparable findings regarding additional cancer detection have been reported in other screening populations with dense breasts. Kelly et al. found that the addition of automated whole-breast ultrasound approximately doubled the cancer detection yield from 3.6 to 7.2 cancers per 1,000 examinations, while also increasing recall examinations [10]. Wilczek et al. likewise reported increased cancer detection when ABUS was added to mammography, although the associated recall rate was considered acceptable within their screening setting [14]. More recently, Jassim et al. performed a systematic review and meta-analysis assessing supplemental ABUS in mammographic screening. The authors concluded that ABUS represents a promising adjunct imaging modality, particularly for women with dense breast tissue, but also highlighted the considerable heterogeneity among the available studies. Differences in study design, patient populations, screening protocols and outcome definitions limited the comparability of results and precluded definitive conclusions regarding the optimal role of ABUS within breast cancer screening strategies [15]. However, most of the available evidence has been generated in women with dense breasts undergoing population-based screening. Data regarding the implementation of ABUS in intensified breast cancer surveillance programs remain limited, and evidence on patient acceptance and workflow integration in this specific setting is scarce. Van Zelst et al. prospectively evaluated the addition of biannual ABUS to annual MRI and mammography in 296 BRCA1/2 mutation carriers. When biannual ABUS was added to annual MRI and mammography, no additional cancers were detected by ABUS. In contrast, the recall rate increased from 8.8% with MRI and mammography to 14.7% when ABUS was added, corresponding to 65 versus 108 recalls among 711 surveillance rounds. The biopsy rate also increased from 6.4% to 7.4%. Thus, in an MRI-based high-risk surveillance setting, the addition of ABUS increased diagnostic work-up without demonstrating incremental cancer detection [16]. These findings underline that the role of ABUS within intensified breast cancer surveillance should be considered in the context of multimodal surveillance rather than as a replacement for MRI. In Germany, intensified surveillance for women at high hereditary risk is based on a multimodality approach that combines annual contrast-enhanced MRI with regular breast ultrasound examinations rather than relying on MRI alone. Women with hereditary breast cancer risk are often young and frequently have dense breast tissue, a population in which ABUS has shown promising results as a supplemental imaging modality [10, 13, 14]. In contrast to van Zelst et al., who primarily addressed the diagnostic value of ABUS within MRI-based surveillance, the present study focused on patient-centered and implementation-related outcomes. From an implementation perspective, the potential role of ABUS can be summarized within a SWOT framework. Strengths include standardized three-dimensional image acquisition, separation of acquisition from physician interpretation, and the absence of ionizing radiation or contrast administration. Weaknesses include relatively long acquisition times and additional interpretation workload. Opportunities include more efficient allocation of physician resources, standardized longitudinal imaging, and future AI-assisted interpretation. Potential threats include additional recalls and biopsies without proven incremental cancer detection, particularly when ABUS is added to MRI-based high-risk surveillance.

Several strengths of this study should be acknowledged. First, the prospective design allowed systematic collection of patient-reported outcomes and workflow parameters. Second, the study was conducted within a certified intensified breast cancer surveillance program, providing real-world evidence from a clinically relevant high-risk population. The study population reflects the actual spectrum of women participating in the GC-HBOC intensified surveillance program, including women with and without a personal history of breast cancer. Accordingly, this heterogeneity reflects routine clinical practice rather than a selectively defined unaffected high-risk cohort. However, findings from the overall cohort should not be interpreted as specifically representative of high-risk women without a previous breast cancer diagnosis. Third, repeated examinations enabled longitudinal assessment of implementation effects over time. The study also has limitations. Its single-center design and voluntary participation may limit the generalizability of the patient-reported acceptance findings. The sample size was moderate, although comparable to other implementation studies in breast imaging. Furthermore, 162 questionnaires were obtained from 73 participants, introducing potential intraindividual correlations despite the use of longitudinal statistical methods. Importantly, the study was not designed to evaluate diagnostic performance and did not include a control group undergoing HHUS alone. Consequently, no conclusions regarding comparative effectiveness or diagnostic superiority can be drawn. Finally, implementation was performed within a certified center of the GC-HBOC providing intensified breast cancer surveillance, and workflow characteristics may differ in other clinical settings. Future studies should further evaluate the long-term workflow effects of ABUS, its role within multimodal surveillance, and the potential impact of AI-assisted interpretation.

## Conclusions

ABUS was associated with high reported patient acceptance and was successfully implemented within the study setting. Examination-related discomfort was minimal, patient satisfaction was high, and willingness to undergo repeat examinations was universal. In addition, implementation was associated with a significant learning effect resulting in increasing workflow efficiency over time. Future studies are warranted to further define the role of ABUS within multimodal intensified breast cancer surveillance programs.

## Data Availability

The data that support the findings of this study are available on request from the corresponding author. The data are not publicly available due to privacy or ethical restrictions.
